# Patterns of claims and determinants of claim rejections in Kuwait's National Health Insurance for Retirees (AFYA): a comprehensive analysis

**DOI:** 10.3389/fpubh.2025.1606980

**Published:** 2025-07-22

**Authors:** Abdullah I. Alibrahim, Hisham Kelendar, Abdulaziz Alhenaidi

**Affiliations:** ^1^Industrial and Management Systems Engineering, Kuwait University, Sabah Al Salem University City, Kuwait City, Kuwait; ^2^Ministry of Health, Kuwait City, Kuwait; ^3^Kennedy School of Government, Harvard University, Cambridge, MA, United States; ^4^GeoHealth Lab, Dasman Diabetes Institute, Kuwait City, Kuwait

**Keywords:** claims data, claim rejections, Kuwait, health insurance, health reform

## Abstract

**Introduction:**

Health insurance claim rejections can impose significant administrative and financial burdens, yet data from emerging national programs are limited. Kuwait's AFYA program, launched in 2016 for retirees, provides a valuable opportunity to study rejection patterns and identify the demographic and service-level factors that influence denial rates in a rapidly evolving high-income context.

**Methods:**

This retrospective study analyzed 4.44 million AFYA claims from 2016 to 2023. Key variables included beneficiary characteristics (age group, sex), provider type, service category (dental, medical, pharmaceutical), claimed amount, and claim year. Logistic regression was employed to determine predictors of claim rejection, controlling for all the above factors. Sensitivity analyses excluded the top 1% of claimed amounts to check for robustness.

**Results:**

The overall rejection rate was 3.85%, lower than reported rates in some established systems. Younger retirees (under 40) had 1.82 times higher odds of claim denial than the reference group (56–60), and female beneficiaries had 1.21 times higher odds than males. Dental services were associated with a 2.28-fold increase in rejections relative to pharmaceutical claims. Laboratory claims, though relatively rare, showed exceptionally high rejection proportions. Rejection rates gradually declined over time, from 4.15% in 2017 to 3.42% by 2023. The most frequent reasons for denial involved uncovered services and insufficient clinical justification.

**Discussion:**

These findings underscore the critical role of clear coverage definitions, consistent coding, and effective administrative oversight in minimizing denials. Younger retirees, female beneficiaries, and certain service types (dental, laboratory) emerged as particularly vulnerable to rejections, indicating the need for targeted policy refinements. Notably, the downward trend in rejections suggests that AFYA has capacity for adaptive improvements over time.

**Conclusion:**

By revealing pivotal factors that drive or mitigate claim rejections, this analysis offers practical guidance for policymakers and healthcare administrators. Standardized electronic forms, provider feedback loops and tighter coverage definitions could trim residual denials without restricting access. AFYA's experience offers transferable lessons for high-income countries seeking to expand private-sector purchasing while containing cost.

## 1 Introduction

Large-scale claims data offer crucial insights into healthcare financing and service delivery, yet in many Gulf Cooperation Council (GCC) countries, these data remain underutilized for guiding policy reforms and improving population health outcomes (1–4). Kuwait illustrates the need and the opportunity for this research: life expectancy at birth has climbed to 82.7 years, but non-communicable diseases already account for around 65% of mortality and diabetes prevalence among adults reaches 25.6% (5–7). Rising longevity, multimorbidity and fiscal pressure therefore intensify the search for delivery models that can preserve equity while containing cost growth.

Kuwait's AFYA program, introduced in 2016, was the first government-sponsored health insurance scheme to augment the Ministry of Health's (MoH's) universal in-kind entitlement by purchasing private insurance to allow supplemental service provision from private providers for retirees and other vulnerable groups. Government transfers to the retiree-insurance line item rose from KD 88.9 million (FY 2018/19) to KD 181.3 million (FY 2023/24), a CAGR of 15.4% (8). Broader MoH spending has also climbed (public share ≥86% of THE). By examining the real-world patterns of claim submissions and denials, AFYA provides a window into how coverage, administrative oversight, and benefit design can facilitate or hinder equitable care in a high-income setting where total health spending per capita is increasing rapidly (9).

Claim rejections constitute a pervasive challenge in health systems worldwide, shaping provider finances, insurer operations, and patient access to timely treatment (10, 11). In Kuwait, public facilities still deliver about 80% of total health spending, yet private-sector engagement is accelerating under the National Development Plan (12). AFYA (Arabic for “wellness”) represents a milestone in Kuwait's healthcare landscape: it seeks to secure comprehensive benefits for retirees while involving private-sector partners in service provision. AFYA is therefore a timely natural experiment in public–private balance within regional and high-income countries. However, high rejection rates can erode trust in such initiatives, delay needed treatments, and undercut public health objectives, especially if at-risk subpopulations are disproportionately affected. Although studies from contexts like the United States suggest denial rates may exceed 14% (10, 13, 14), evidence from emergent programs in the GCC remains scarce (15–18).

Established through Law No. 114 of 2014 (19), AFYA initially insured ~105,000 retirees—roughly 2.5% of Kuwait's population (8, 20). Over time, eligibility expanded to women under public aid, widows, and divorced women, eventually encompassing 200,000 enrollees by 2023 (activated in 2024) (8, 21, 22). This publicly funded yet privately managed insurance was envisioned as a cornerstone of broader public-private integration in healthcare, aligning with the Kuwait National Development Plan (8, 23). Parliamentary transcripts cite three goals beyond cost control: (i) reducing public-hospital waiting times, (ii) freeing MoH capacity, and (iii) stimulating private investment in diagnostics and ancillary services.^1^

Separate from the AFYA health insurance program, Kuwait operates a classic contributory, defined-benefit social-insurance model administered by the Public Institution for Social Security (PIFSS). PIFSS is a pay-as-you-go, defined-benefit pension scheme (Law 61/1976) (25). Contribution rates are 7.5% employee, 11% employer, 10% Treasury for civilians, replacement rates often > 80% (26, 27). PIFSS covers cash risks (old-age, disability, death, maternity leave, unemployment, work injury). No medical benefits are paid from PIFSS. Healthcare for citizens—including retirees—is financed in-kind by MoH; AFYA merely outsources some of that care to private providers since 2016, operated by Gulf Insurance Group. Premiums are paid straight out of the MoH budget and have no actuarial link to PIFSS pension funds.^2^

In this paper, we analyze 4.44 million AFYA claims to illuminate how administrative oversight, coverage criteria, and demographic factors influence denials. Specifically, we (1) describe overall claim submission and rejection patterns; (2) identify beneficiary, provider, and service-level determinants of rejections; and (3) assess temporal shifts as AFYA expanded its beneficiary base. By leveraging this comprehensive dataset, we contribute evidence on how emerging national schemes can refine coverage definitions and reduce systemic barriers to care. Our findings have immediate relevance not only for Kuwait but also for comparable high-income nations that seek to strike a balance between cost containment, financial sustainability, and equitable healthcare provision.

## 2 Methods

### 2.1 Study design

We conducted a retrospective analysis of health insurance claims drawn from Kuwait's AFYA program spanning October 1, 2016, to December 31, 2023. This design captures all claims submitted during the full operational window of AFYA, providing a comprehensive view of emerging claim patterns over time. The AFYA program, as previously described, served as a supplemental national health insurance scheme initially targeting Kuwaiti retirees and later expanding to encompass additional at-risk groups.

### 2.2 Data source and study population

The study population included all AFYA enrollees who submitted claims during the specified timeframe. Initially encompassing ~105,000 Kuwaiti retirees in 2016, eligibility criteria broadened over time to reach nearly 200,000 enrollees by 2023. A total of 4,437,503 claims were analyzed, allowing a comprehensive examination of claim dynamics without necessitating sampling.

The dataset was provided by the Health Insurance Department at the Ministry of Health, Kuwait, under a data-sharing agreement ensuring adherence to confidentiality standards.

Ethical approval for this study was obtained from the Permanent Committee for Research Ethics of the MoH, Kuwait (Approval Number 2288/2023). All procedures aligned with the principles outlined in the Declaration of Helsinki, and patient privacy was safeguarded through anonymization of all personally identifiable information.

### 2.3 Variables and coding

We analyzed demographic factors (e.g., age, sex), claim-level attributes (e.g., requested amount, type of service, care setting), and temporal indicators (year of claim). “Rejected” or “Accepted” status was the primary outcome. To standardize free-text information, we categorized provider types into “hospital,” “clinic,” “pharmacy,” “lab,” or “other,” and clinical services into “dental,” “medical,” or “pharmaceuticals” based on procedure descriptions.

All claims are submitted through the insurer's electronic portal, which enforces hard “must-fill” rules for beneficiary ID, service date, provider ID, service category, and claimed amount; a claim cannot be lodged until these fields pass built-in format and range checks. All variables underwent thorough cleaning and validation to ensure data quality and consistency. As a result of the portal-level validation, and our own post-extract completeness check, no claims had missing or incomplete key variables. We then applied additional quality controls: (i) logical-consistency checks (e.g., service date within coverage period, amount ≥ 0); (ii) duplicate detection on the composite key of claim, date, and provider; and (iii) outlier screening, flagging observations above the 99.9th percentile of claimed amount for manual review. No records failed the logical checks, and outlier claims were retained after confirmation that they reflected legitimate high-cost episodes. When possible, data and variable interpretations were verified against administrative records by a team at the MoH. All available variables were used, except procedure codes and provider names were categorized as described in the following list of variables.

The outcome of interest was claim status, categorized as “Accepted” or “Rejected.” Key predictor variables were selected based on their theoretical and policy relevance. These included:

- Patient-level characteristics: age group in 5-year intervals [e.g., “(051–055),” “(056–060)”], sex (male/female).
- Claim-level characteristics: claimed amount (in Kuwaiti Dinars), and service setting (e.g., outpatient or inpatient).
- Temporal indicators: year and month of authorization.
- Utilization and benefit-related factors: indicators such as whether the claim would exceed the predefined coverage limit (*exceed limit*: yes/no), service type (medical, dental, pharmaceutical), and facility filing the claim (clinic, hospital, pharmacy, lab, other).
- Rejection-specific information: rejection categorization (e.g., “Exclusion”) and rejection reason code.

To facilitate meaningful analyses, certain variables were coded from free-text available variables as follows:

- Provider filing claim: the *authorized provider* variable was used to classify facilities into *hospital, clinic, lab, pharmacy*, or *other* based on keyword pattern matching (e.g., “hospital” for hospital facilities).
- Service type: the *procedure type* variable was transformed into “Dental,” “Medical,” “Pharmaceuticals,” or “Other” categories to streamline analyses across diverse coding schemes.
  The dataset and variables review process was carried out in collaboration with the Insurance Department of the Ministry of Health.

### 2.4 Statistical analysis

We used descriptive statistics to characterize the distribution of claims and examined the prevalence of rejection across patient, provider, and clinical attributes. The continuous variable *claimed amount* was assessed for normality, and then standardized (*z*-scores). Given that claimed amounts were skewed, nonparametric tests (e.g., Wilcoxon rank-sum tests) were employed to compare medians between accepted and rejected claims. This choice was made to avoid assumptions of normality and to provide robust estimates of distributional differences (28).

To screen individual predictors against claim status (bivariate analysis), we first conducted associations between categorical predictors (e.g., sex, service category) and claim status using chi-square tests. These tests were strictly bivariate and served only as descriptive exploration. Next, we fitted a multiple logistic regression model with claim status as the dependent variable. Logistic regression was chosen for its suitability in estimating the odds of binary outcomes, adjusting for multiple covariates, and providing interpretable effect measures (odds ratios). Calendar year was entered as a set of fixed-effect dummies rather than a single linear trend term because policy shocks during the study period were non-linear [e.g., the benefit expansion in 2019 and the eligibility expansion approved in 2023 but activated in 2024 (8)]. For categorical variables, the reference category for each variable was set to the mode category (most frequent) to produce meaningful odds ratios. Rare categories (<1% of observations) were combined with conceptually similar groups to avoid sparse-cell bias and improve interpretability. All covariates described above were entered simultaneously into the multivariable model to estimate their independent effects. The statistical analyses were conducted using R Software v4.4.2 (2024-10-31) (29).

#### 2.4.1 Model diagnostics and validation

To verify that the multivariable logistic model met key assumptions and produced reliable estimates, we implemented a structured set of post-estimation diagnostics. First, multicollinearity was assessed using Variance Inflation Factors (VIFs); all VIFs were below 4, indicating negligible collinearity among predictors (30). Model goodness-of-fit was examined with the Hosmer–Lemeshow test (*g* = 10 risk deciles) and three pseudo-*R*^2^ indices (McFadden, Cox–Snell, and Nagelkerke) (31).

Overall discrimination was quantified by the area under the receiver-operating characteristic curve (AUC), while a decile-based calibration plot contrasted observed vs. predicted rejection probabilities to check agreement across risk strata (32).

Because this is an exploratory analysis aimed at generating policy-relevant insights rather than building a definitive predictive tool, we did not include interaction terms. Omitting higher-order effects reduces the risk of overfitting, simplifies interpretation, and aligns with best-practice recommendations for initial explanatory modeling in large administrative datasets (33).

### 2.5 Sensitivity and robustness analyses

Sensitivity analyses were conducted to evaluate the robustness of the findings. Claims with extraordinarily high claimed amounts (top 1%) were considered potential outliers. After excluding these outliers, analyses were repeated to ascertain whether they disproportionately influenced the results. In addition, subgroup analyses by age group and provider types were performed to determine whether observed patterns held across different patient demographics and facility categories. Similar approaches have been employed in health services research to ensure stable and reliable estimates (34).

## 3 Results

### 3.1 Overall claims and demographic patterns

A total of 4,437,503 claims were analyzed, of which nearly half (47.5%) were attributed to female beneficiaries (Table 1). The most common age group was (51–55), which accounted for 20.5% of the claims. Year-by-year, there was a steady increase in the number of claims; increasing from 484,838 in 2017 to 815,594 in 2023.

**Table 1:** Profile of AFYA claims.

**Variable**	**Overall** **(*N* = 4,437,503)^a^**	**Approved** **(*N* = 4,266,804)^a^**	**Rejected** **(*N* = 170,699)^a^**	***p*-value^b^**
**Age group (years)**				<0.001
Under 40	80,521 (1.8%)	75,167 (1.8%)	5,354 (3.1%)	
41–45	263,077 (5.9%)	249,695 (5.9%)	13,382 (7.8%)	
46–50	587,406 (13%)	561,521 (13%)	25,885 (15%)	
51–55	911,192 (21%)	876,396 (21%)	34,796 (20%)	
56–60	885,191 (20%)	852,766 (20%)	32,425 (19%)	
61–65	700,080 (16%)	675,494 (16%)	24,586 (14%)	
66–70	477,970 (11%)	461,607 (11%)	16,363 (9.6%)	
71–75	277,769 (6.3%)	268,510 (6.3%)	9,259 (5.4%)	
76–99	254,122 (5.7%)	245,478 (5.8%)	8,644 (5.1%)	
100+	175 (<0.1%)	170 (<0.1%)	5 (<0.1%)	
**Sex**				<0.001
Female	2,106,417 (47%)	2,016,642 (47%)	89,775 (53%)	
Male	2,331,086 (53%)	2,250,162 (53%)	80,924 (47%)	
**Provider type**				<0.001
Clinic	633,113 (14%)	604,577 (14%)	28,536 (17%)	
Hospital	2,766,250 (62%)	2,658,748 (62%)	107,502 (63%)	
Laboratory	7,633 (0.2%)	5,595 (0.1%)	2,038 (1.2%)	
Other	451,406 (10%)	435,566 (10%)	15,840 (9.3%)	
Pharmacy	579,101 (13%)	562,318 (13%)	16,783 (9.8%)	
**Service category**				<0.001
Dental	165,823 (3.7%)	155,332 (3.6%)	10,491 (6.1%)	
Medical	1,937,263 (44%)	1,853,753 (43%)	83,510 (49%)	
Other	206 (0.00%)	2 (0.00%)	204 (0.00%)	
Pharmaceuticals	2,334,211 (53%)	2,257,717 (53%)	76,494 (45%)	
**Care setting**				<0.001
Inpatient	257,820 (5.8%)	249,809 (5.9%)	8,011 (4.7%)	
Outpatient	4,179,477 (94%)	4,016,993 (94%)	162,484 (95%)	
**Exceeds limit**	365,045 (8.2%)	194,346 (4.6%)	170,699 (100%)	<0.001
**Requested amount (KWD)**	24 (9, 120)	24 (9, 120)	26 (9, 110)	0.2
**Year**				<0.001
2016	93,892 (2.1%)	89,997 (2.1%)	3,895 (2.3%)	
2017	484,838 (11%)	459,832 (11%)	25,006 (15%)	
2018	544,186 (12%)	518,735 (12%)	25,451 (15%)	
2019	568,972 (13%)	543,677 (13%)	25,295 (15%)	
2020	535,372 (12%)	515,962 (12%)	19,410 (11%)	
2021	671,851 (15%)	649,619 (15%)	22,232 (13%)	
2022	722,798 (16%)	701,257 (16%)	21,541 (13%)	
2023	815,594 (18%)	787,725 (18%)	27,869 (16%)	

Summary of claims data from Kuwait's AFYA program, including demographic, procedural, and provider-related variables. The table compares approved vs. rejected claims and highlights significant differences across key attributes.

a *n* (%); Median (Q1, Q3).

b Pearson's Chi-squared test; Wilcoxon rank sum test.

### 3.2 Claim characteristics and patterns of rejection

Claims covered a broad spectrum of services (Table 1). Hospitals submitted most of the claims, growing from 58.7% of all claims in 2016 to 66.7% by 2023. Importantly, the “hospital” billing category includes not only inpatient stays but also outpatient encounters and prescription claims delivered on hospital premises; hence most hospital-filed claims are outpatient or pharmacy, with some inpatient stays. Although hospitalizations only represented 5%–6% of the total claim count, they contributed ~35% of the total amount claimed in Kuwaiti Dinars (KWD). Outpatient services consistently dominated the volume of claims, averaging around 94% of all submissions each year.

By service category, pharmaceutical claims were the most common (52.6% of claims), followed by medical services (43.7%), and dental procedures (3.7%). It was interesting to note that dental procedures increased from 2.1% in 2017 to 4.2% in 2023. Claims exceeding predefined coverage limits fluctuated between 7.2% and 9.5% without a clear temporal trend. Overall, 3.85% of claims were rejected. The median claimed amounts did not differ significantly between accepted claims (23.70 KWD; IQR 111.36) and rejected claims (25.60 KWD; IQR 101.22) (Wilcoxon rank-sum, *p* = 0.20). Among provider types, claims from hospitals had a rejection rate of 3.89%, clinics 4.51%, pharmacies 2.90%, and labs 26.70%, suggesting that laboratories, albeit representing <1% of all submissions, were associated with notably higher rejection rates.

### 3.3 Temporal trends in claim submissions and rejections

Across the study period, the annual volume of submitted claims increased from 484,838 in 2017 to 815,594 in 2023. Despite this growth, overall rejection rates exhibited a modest decline, dropping from 4.15% in 2017 to 3.42% in 2023 (Table 1). The sharpest decreases occurred after 2020, coinciding with AFYA's policy expansions.

**Figure 1:**
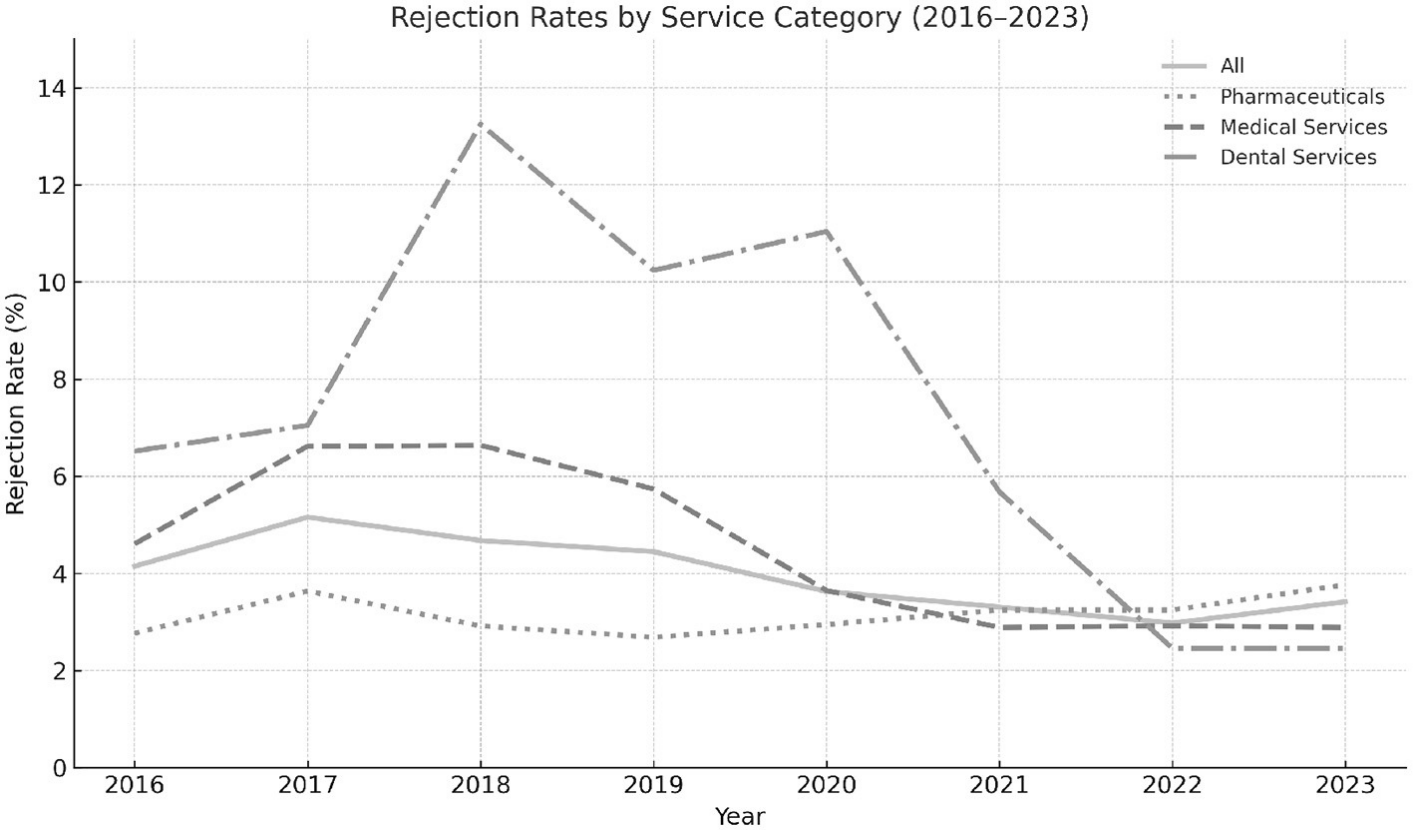
Trends in annual claim rejection rates across all service types, including pharmaceuticals, medical services, and dental procedures, within Kuwait's AFYA program. The figure highlights variations in rejection patterns over time and between service categories.

As illustrated in Figure 1, rejection rates varied by service category. Dental services observed notably higher and more volatile rates during the early years, peaking near 14% before converging toward the overall average by the end of the study period. By contrast, pharmaceuticals and medical services maintained comparatively lower and more stable rejection rates throughout. These patterns highlight the differential impacts of benefit design, clinical complexity, and provider-type heterogeneity.

**Figure 2:**
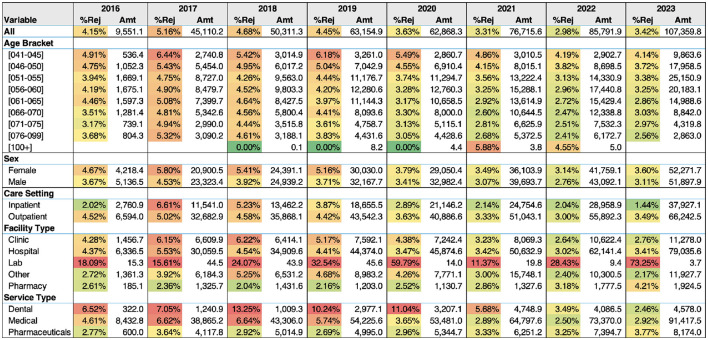
A heatmap showing rejection rates (%Rej) over time for each subgroup along with the amount claimed (Amt) in thousands of Kuwaiti Dinars for each subgroup during the year.

Further disaggregation of rejections by demographic and institutional factors, presented in Figure 2, reveals considerable heterogeneity across age groups, care settings, and facility types. Younger age beneficiaries, outpatient services, and standalone laboratories were initially associated with higher rejection rates. Over time, however, these disparities narrowed.

### 3.4 Factors associated with claim rejection

Chi-square tests revealed significant differences (*p* < 0.001) in rejection proportions across key strata, including sex, service category, and provider type. In unadjusted comparisons, female beneficiaries exhibited slightly higher rejection rates than males, and dental or medical claims demonstrated greater rejection rates than pharmaceuticals, particularly in the early years of AFYA's implementation.

### 3.5 Logistic regression findings

Multiple logistic regression analysis (Table 2) further explored these associations. Beneficiaries < 40 years had 82% higher odds of claim denial than the reference group (56–60 years) (OR 1.82, 95% CI 1.76–1.87); the odds progressively declined in older age bands. Female beneficiaries were also at increased risk (OR 1.21, 95% CI: 1.20–1.23). Relative to clinic-submitted claims, those filed by providers classified as “Other” (predominantly stand-alone laboratories) had lower odds of rejection (OR 0.77, 95% CI 0.75–0.78), while hospital-filed claims showed odds similar to clinics (OR 0.99, 95% CI 0.98–1.00). When service category was considered, dental services exhibited more than double the odds of rejection compared with the reference category (“Pharmaceuticals”) (OR 2.28, 95% CI 2.22–2.33), and medical services also carried elevated odds (OR 1.32, 95% CI 1.30–1.33). Outpatient encounters were 53% more likely to be rejected than inpatient stays (OR 1.53, 95% CI 1.50–1.57). Each one-standard-deviation increase in the claimed amount was associated with a 4% rise in the odds of rejection (OR 1.04, 95% CI 1.03–1.04). Finally, compared with 2020 (reference year), odds were significantly higher in 2016–2019 and lower in 2021–2023, reflecting recent policy adjustments.

**Table 2:** Adjusted odds of claim rejection.

**Variable**	**Odds ratio** **(*N* = 4,437,503)**	**95% CI** **(lower, upper)**	***Z*-statistic**	***p*-value**
**Age group (years)**				
Under 40	1.82	(1.76, 1.87)	38.88	<0.001
41–45	1.38	(1.35, 1.41)	30.59	<0.001
46–50	1.20	(1.18, 1.22)	21.31	<0.001
51–55	1.04	(1.02, 1.06)	5.05	<0.001
56–60	Ref.	–	–	–
61–65	0.95	(0.94, 0.97)	–5.52	<0.001
66–70	0.94	(0.92, 0.96)	–6.41	<0.001
71–75	0.93	(0.91, 0.96)	–5.68	<0.001
76–99	0.99	(0.97, 1.01)	–0.78	0.436
100+	0.99	(0.35, 2.17)	–0.02	0.988
**Sex**				
Female	1.21	(1.20, 1.23)	38.51	<0.001
Male	Ref.	–	–	–
**Provider type**				
Clinic	Ref.	–	–	–
Hospital	0.99	(0.98, 1.00)	–1.31	0.191
Other	0.77	(0.75, 0.78)	–26.35	<0.001
Pharmacy	0.83	(0.81, 0.84)	–17.94	<0.001
**Service category**				
Dental	2.28	(2.22, 2.33)	68.15	<0.001
Medical	1.32	(1.30, 1.33)	47.69	<0.001
Pharmaceutical	Ref.	–	–	–
**Care setting**				
Inpatient	Ref.	–	–	–
Outpatient	1.53	(1.50, 1.57)	33.47	<0.001
**Amount (scaled)**	1.04	(1.03, 1.04)	14.93	<0.001
**Year**				
2016	1.11	(1.07, 1.15)	5.76	<0.001
2017	1.44	(1.41, 1.46)	36.84	<0.001
2018	1.32	(1.30, 1.35)	28.53	<0.001
2019	1.23	(1.20, 1.25)	20.99	<0.001
2020	Ref.	–	–	–
2021	0.91	(0.90, 0.93)	–8.91	<0.001
2022	0.82	(0.80, 0.84)	–19.74	<0.001
2023	0.89	(0.87, 0.90)	–12.40	<0.001

Results of logistic regression analysis showing the adjusted odds ratios (OR) and confidence intervals (CI) for factors associated with claim rejections, including demographic characteristics, provider types, and procedure categories.

Ref, reference category.

In terms of service categories (taking *pharmaceutical* as reference), *dental* claims were associated with more than double the odds of rejection (OR 2.28, 95% CI: 2.22–2.33), and claims coded as *medical* also showed increased odds (OR 1.32, 95% CI: 1.30–1.33). Outpatient care setting was associated with higher odds of rejection compared to inpatient care (OR 1.53, 95% CI: 1.50–1.57). The scaled claimed amount was associated with slightly higher odds of rejection (OR 1.04, 95% CI: 1.03–1.04), suggesting that more expensive claims were somewhat more likely to be denied. Furthermore, using 2020 as the baseline year, claims in 2016–2019 all had higher odds of rejection (OR range: 1.11–1.44), whereas those from 2021–2023 showed lower odds (OR range: 0.82–0.91), indicating a temporal trend toward fewer rejections in more recent years.

#### 3.5.1 Model diagnostics

Consistent with the exploratory aim of this study—to identify factors associated with claim rejection rather than to build a high-performance prediction tool—we focused on checking major specification errors that could distort effect estimates. All variance-inflation factors were comfortably below the accepted threshold of 5 [GVIF^1/(2·*df*)^ range: 1.00–1.11; Supplementary Table S3], confirming the absence of problematic multicollinearity.

The Hosmer–Lemeshow goodness-of-fit test was statistically significant [$\chi_{\left(8\right)}^{2}=1,856,p<0.001$]. Given the very large sample size (>4 million claims), even trivial deviations from perfect calibration can trigger significance. Global measures of explained variation were modest (McFadden *R*^2^ = 0.017; Nagelkerke *R*^2^ = 0.020), and the model's discriminatory ability was limited (AUC = 0.60). These values are typical for exploratory administrative-claims analyses in which many unmeasured workflow factors influence adjudication. Crucially, however, the diagnostics did not reveal violations that would bias odds-ratio estimates, supporting the model's suitability for hypothesis generation and policy insight.

### 3.6 Exploring reported rejection reasons

Over the study period, the most frequently cited reasons for claim rejections exhibited notable consistency, with a few key categories repeatedly emerging at the top. Specifically, reasons related to services or conditions not covered under policy terms, requests for tests or treatments not justified by the stated diagnosis, and limitations involving aging conditions and related treatments were among the most prevalent. While some reasons, such as tumor-related exclusions or the denial of treatments deemed cosmetic or unrelated to the claimed condition, appeared intermittently, the majority of recurring factors pointed to gaps in policy coverage comprehension and clinical justification. For a detailed breakdown of the top five reported rejection reasons by year, please refer to Supplementary Material A.

### 3.7 Sensitivity analysis

Excluding claims within the top 1% of requested amounts did not materially change the key findings; the adjusted odds ratios of all major predictors remained within 5% of their original estimates. Subgroup analyses stratified by age group and provider type also reinforced the main results, indicating that the identified predictors of rejection were not concentrated within a single demographic or organizational subgroup. This consistency supports the robustness of the observed patterns. Results of the logistic regression model conducted for the sensitivity analysis are shown in Supplementary Material B.

## 4 Discussion

This is the first population-level assessment of claim denials in any GCC social insurance program. The study provides the a comprehensive look at claim rejections within Kuwait's AFYA program, highlighting macro-level trends in claims and granular predictors of rejection. Because AFYA is restricted to Kuwaiti citizens, foreigners resident in Kuwait obtain coverage either through the employer-based “Dhaman” scheme, private insurance, or sponsor-paid contributions to MoH expat assurance program; they therefore lie outside the present analytic frame (12). Although the overall AFYA claims denial rate (3.85%) is relatively low compared to mature systems like the United States [up to 14% (10)], administrative burdens can still be substantial given AFYA's high claim volume of over 4 million claims submitted since inception. Below, we discuss the results through four thematic lenses: (1) administrative and provider-level factors, (2) beneficiary-level factors, (3) temporal trends and potential policy shifts, and (4) comparisons with other systems and implications for policy.

### 4.1 Administrative and provider-level factors

A key finding concerns the distinct rejection patterns across provider types. Although hospitals, clinics, and pharmacies had relatively modest rejection rates (3.89%, 4.51%, and 2.90%, respectively), standalone laboratories experienced rejections for more than one in four claims (26.70%). It is important to note only 0.2% of all claims came from independent laboratories. Still, findings confirm that growing vertical integration, where diagnostic services are housed within larger healthcare systems, appears to reduce administrative complexity and streamline reimbursement processes.

High rejection rates for dental procedures also emerged as an important issue, with more than double the odds (OR = 2.28) of rejection relative to pharmaceuticals. Initially designed for retirees and chronic condition management, the AFYA program did not appear to fully anticipate the demand for comprehensive dental care. Although expansions in coverage (e.g., crowns and implants) reduced rejections in later years, trends highlight how benefit designs evolve alongside beneficiary needs.

Although most services require prior approval from the insurance company before being provided, the beneficiary can complain to the insurance company to clarify the reason for rejection. If not satisfied, the beneficiary can complain to the Health Insurance Directorate within the MOH, where the medical team will review the case and the cause of rejection. The insurance company will then be obliged to provide the service or refund the beneficiary if the rejection reason was not justified. Furthermore, the medical team within the Health Insurance Directorate regularly reviews rejections and complaints, including those submitted to the insurance company.

Our findings do not support the notion of blanket exclusions of expensive care. Our fully adjusted model—which includes claim amount as a continuous covariate—shows only a marginal increase in denial odds for high-cost claims (OR = 1.04, 95% CI 1.03–1.04). Moreover, “service outside benefit package” and “insufficient clinical justification” were the dominant rejection codes, together accounting for < 15% of all denials. These findings suggest that AFYA's cost-control strategy relies on medical-necessity filters rather than price ceilings, indicating no systemic exclusion of costly but clinically indicated treatments.

### 4.2 Beneficiary-level factors

Age-related variations underscore how certain demographics are at higher risk of claim rejections. Compared to the reference age group of 56–60 years, younger beneficiaries (e.g., those under 40) were 82% more likely to have their claims denied (OR = 1.82). This finding likely reflects heightened scrutiny of services perceived as elective or insufficiently justified under current coverage definitions. This scrutiny can be explained by the preconception that most services provided to beneficiaries in this category are elective. This preconception may be driven by the fact that most beneficiaries are above 50 years old, and the AFYA policy primarily targets retirees and older women. Another explanation is that retirees at younger ages might be medically retired due to specific diseases, such as genetic or hereditary diseases, which are excluded from the coverage of the AFYA policy.

Conversely, older enrollees, initially the program's core beneficiaries, showed lower odds of rejection (e.g., OR = 0.94 for ages 66–70). These differences persist even after we control for service mix and claim amount, and they do not reflect sampling bias: the dataset covers the *entire* universe of 4.44 million AFYA claims from 2016 to 2023. Although mild, but heterogeneity may arises because of benefits expansions in 2019. Robustness checks that (i) removed the top 1% of claim amounts and (ii) collapsed five-year age bands yielded substantively unchanged coefficients, supporting internal homogeneity of the analytic sample.

Sex differences also emerged; claims for female beneficiaries had 21% higher odds of rejection than males (OR = 1.21). This gap may be driven by potential differences in the service mix. The causes of rejection reported were that services, investigations, and treatments are not covered by the policy, such as treatment and investigations for congenital diseases, cosmetic treatments, medical devices or appliances, and weight loss treatments. In addition, causes related to inpatient stay include cases that are not justified for admission or extension of ongoing admission. Other causes of rejection included services that were not related to or not justified by the diagnosis and submitted investigation within normal limits, and did not warrant further investigation. Tailored educational initiatives (e.g., workshops or provider bulletins) that clarify coverage boundaries for beneficiaries across different age groups and service types could help reduce these disparities and ensure more consistent claims submission and approval.

### 4.3 Temporal trends and potential policy shifts

Rejection rates declined modestly over time, dropping from 4.15% in 2017 to 3.42% by 2023. This downward trend coincided with AFYA's expansion to broader demographic groups and may reflect an organizational learning curve wherein both administrative staff and beneficiaries adapted to the program's requirements. The modest decrease in rejection rates is accompanied by persistent rejection justifications. For example, the most frequently cited reasons for denials (services outside policy scope, treatments not justified by diagnosis, and exclusions of aging conditions) endured for the majority of the program's existence. Addressing these challenges via clearer benefit definitions and streamlined claim review protocols (e.g., standardized digital forms, real-time adjudication software) can further reduce rejection rates. Policymakers in Kuwait might also consider pilot-testing a feedback system in which providers receive regular, detailed reports on denial reasons, enabling targeted improvements in documentation and billing practices.

### 4.4 Comparisons with other systems and implications for policy

Although Kuwait's overall rejection rate is comparatively modest, the program's large claim volume, exceeding 815,000 submissions in 2023, magnifies the administrative challenges of even a 3%–4% rejection rate. Similar to experiences in the United States, where providers lose 3%–5% of net revenues to claim denials (14), such inefficiencies can accrue substantial costs over time. Ireland's delayed rollout of universal insurance (35) demonstrates how ambitious coverage expansions must be backed by robust administrative frameworks, including standardized coding, clearly defined benefit packages, and stable funding mechanisms (36).

Kuwait benefits from a centralized claims database, yet more extensive provider-level data integration and standardized clinical coding (e.g., ICD-10) would enhance policy utility. Trust in health insurers, exemplified by concerns in the Netherlands (37), similarly highlights the need for transparent claims processes, prompt resolution of appeals, and clear communication of policy limits. Taken together, Kuwait's experience underscores how data-driven oversight, along with iterative refinements to coverage policy and submission protocols, can fortify the sustainability and equity of national health insurance schemes in high-income contexts.

### 4.5 Limitations and future research

This study's conclusions should be weighed against certain data limitations. The absence of unique patient identifiers prevents discerning repeat claims or measuring individual-level trajectories. Free-text entry of diagnoses and procedures hampers granular analysis of clinical appropriateness. Future research or policy-led improvements—such as linking claims with electronic health records, establishing a robust patient ID system, and mandating use of standardized coding—would address these gaps. Furthermore, the scope of rejection reasons relies on provider and insurer coding accuracy, which may occasionally mask deeper systemic issues such as fraud or abuse. Future research incorporating patient-level linkages and standardized diagnostic coding could enable more detailed analyses of patient pathways and clinical outcomes, further enhancing the program's evaluative framework.

Although this study did not stratify claims by geriatric or palliative care services, Kuwait's rapidly aging population—combined with rising demand for long-term and end-of-life care—calls for future evaluations that explicitly examine utilization and cost patterns among older adults. As life expectancy continues to rise, tailored benefit designs and service delivery models for older adults will become increasingly central to the sustainability and equity of insurance programs like AFYA.

In sum, Kuwait's AFYA program presents a microcosm of the challenges and opportunities facing emerging national insurance schemes in high-income contexts. While the overall rejection rate is relatively modest, the persistent administrative friction among younger beneficiaries, certain service categories (e.g., labs, dental), and specific rejection reasons necessitates continuous policy refinements. Strengthening coverage guidelines, standardizing coding, investing in training, and enhancing administrative oversight offer practical steps to bolster efficiency, reduce administrative waste, and safeguard provider sustainability. These approaches are relevant to Kuwait and other high-income nations pursuing similar reforms to balance cost containment with equitable coverage for older and vulnerable populations.

## 5 Conclusion

This study provides the first in-depth assessment of claims and rejection patterns under Kuwait's AFYA program, revealing how administrative processes, coverage rules, and beneficiary demographics intersect to shape care access. Although the program's overall denial rate (3.85%) is lower than in some established systems, AFYA's large claim volume magnifies the adverse effects of even a modest rejection proportion—particularly among younger retirees, female beneficiaries, and specific services like dental and laboratory tests. These findings highlight how incomplete coverage definitions and inconsistent coding or billing practices can become systemic barriers to care, underscoring the necessity of more robust administrative oversight and policy alignment.

From a health economics perspective, AFYA's experience exemplifies the trade-offs involved in scaling up private-sector engagement and navigating policy expansions in a high-income environment. Standardizing coding procedures, refining benefit packages based on evidence, and adopting real-time claim adjudication systems may diminish rejection rates and enhance fairness in resource allocation. Moreover, transparent feedback loops—whereby providers receive detailed and timely denial rationales—could bolster both provider compliance and patient trust. As policymakers in Kuwait evaluate AFYA's performance and consider broader reforms, our findings emphasize the importance of a data-driven approach that empowers decision-makers to target critical points of friction.

These insights are not limited to Kuwait. High-income nations worldwide are grappling with growing demand for inclusive coverage, escalating healthcare expenditures, and the need to protect vulnerable populations from financial risk. By shedding light on administrative bottlenecks and demographics at risk of denial, this study contributes to international debates on how to achieve efficiency, equity, and sustainability in health insurance models. Addressing coverage ambiguities, improving administrative coordination, and aligning benefit design with population needs remain pivotal steps to ensure that evolving national insurance programs truly advance public health goals.

## Data Availability

The data analyzed in this study is subject to the following licenses/restrictions. The datasets analyzed for this study are available from the authors on reasonable request, subject to necessary approvals. Requests to access these datasets should be directed to Insurance Department, Ministry of Health, Kuwait.

## Footnotes

^1^For further details on Kuwait's healthcare system, the development of the AFYA program, and public attitudes toward health insurance reforms, we recommend the following sources: Mossialos et al. (12), Alibrahim et al. (8), Alnashmi et al. (24).

^2^Throughout this paper, “AFYA” refers to the retiree health-insurance program established under Law 114/2014 and funded from the MoH budget; it is entirely separate from PIFSS, the contributory pension system created by Law 61/1976.
