# Supplementary material for: Patterns of claims and determinants of claim rejections in Kuwait's National Health Insurance for Retirees (AFYA): a comprehensive analysis
Source: Front Public Health. 2025 Jul 22;13:1606980. doi: 10.3389/fpubh.2025.1606980 (PMC12321863; doi:10.3389/fpubh.2025.1606980)
Supplement: Supplementary file 1 [file Data_Sheet_1.pdf]

Supplementary Material

1 SENSITIVITY ANALYSIS ON LOGISTIC REGRESSION

Table S1. Adjusted odds ratios for claim rejection after excluding claims in the top 1% of requested amounts, demonstrating the robustness of the primary findings.

Variable	Odds Ratio (N = 4,437,503)	95% CI (Lower, Upper)	Z-statistic	p-value
**Age Group (years)**				
Under 40	1.82	(1.76, 1.87)	38.88	<0.001
41–45	1.38	(1.35, 1.41)	30.59	<0.001
46–50	1.20	(1.18, 1.22)	21.31	<0.001
51–55	1.04	(1.02, 1.06)	5.05	<0.001
56–60	Ref.	–	–	–
61–65	0.95	(0.94, 0.97)	-5.52	<0.001
66–70	0.94	(0.92, 0.96)	-6.41	<0.001
71–75	0.93	(0.91, 0.96)	-5.68	<0.001
76–99	0.99	(0.97, 1.01)	-0.78	0.436
100+	0.99	(0.35, 2.17)	-0.02	0.988
**Sex**				
Female	1.21	(1.20, 1.23)	38.51	<0.001
Male	Ref.	–	–	–
**Provider Type**				
Clinic	Ref.	–	–	–
Hospital	0.99	(0.98, 1.00)	-1.31	0.191
Other	0.77	(0.75, 0.78)	-26.35	<0.001
Pharmacy	0.83	(0.81, 0.84)	-17.94	<0.001
**Service Category**				
Dental	2.28	(2.22, 2.33)	68.15	<0.001
Medical	1.32	(1.30, 1.33)	47.69	<0.001
Other	Ref.	–	–	–
**Care Setting**				
Inpatient	Ref.	–	–	–
Outpatient	1.53	(1.50, 1.57)	33.47	<0.001
**Amount (scaled)**	1.04	(1.03, 1.04)	14.93	<0.001
**Year**				
2016	1.11	(1.07, 1.15)	5.76	<0.001
2017	1.44	(1.41, 1.46)	36.84	<0.001
2018	1.32	(1.30, 1.35)	28.53	<0.001
2019	1.23	(1.20, 1.25)	20.99	<0.001
2020	Ref.	–	–	–
2021	0.91	(0.90, 0.93)	-8.91	<0.001
2022	0.82	(0.80, 0.84)	-19.74	<0.001
2023	0.89	(0.87, 0.90)	-12.40	<0.001

Ref = Reference Category

2 LOGISTIC REGRESSION ODDS RATIO VISUALIZATION

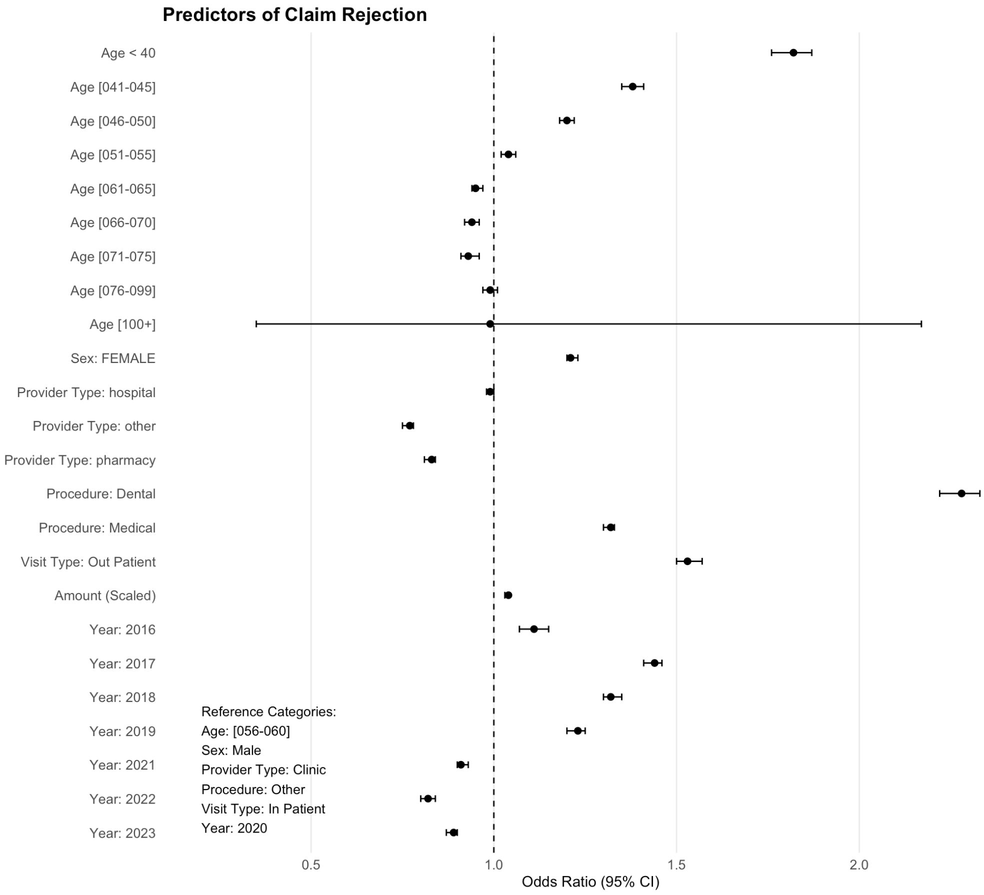

Figure S1. A visual representing the odds ratios of individual covariates with the reference categories shown in the bottom left.

3 MODEL DIAGNOSTICS AND GOODNESS-OF-FIT

3.1 Multicollinearity Check

Table S2. Generalised variance-inflation factors (GVIFs). All adjusted $\mathrm{GVIF}^{1/(2\cdot\mathrm{Df})}$ values are well below the conventional cut-off of 5, indicating negligible multicollinearity.

Predictor	GVIF	Df	$\mathrm{GVIF}^{1/(2\cdot\mathrm{Df})}$
`Procedure Category`	1.54	2	1.11
`Provider Type`	1.45	4	1.05
`Visit Type`	1.21	1	1.10
`Amount Scaled`	1.21	1	1.10
`Year`	1.07	7	1.01
`Age Bracket`	1.06	9	1.00
`Sex`	1.03	1	1.02

3.2 Global Pseudo-R^2 Indices

Table S3. Pseudo-R^2 measures for the full logistic model. Values were produced with `pscl::pR2()` (global indices) and `performance::r2_nagelkerke()` (Nagelkerke).

Statistic	Value
Log-likelihood (model)	-7.1079×10^5
Log-likelihood (null)	-7.2284×10^5
Likelihood ratio G^2	2.4099×10^4
McFadden R^2	0.0167
ML (McKelvey–Zavoina) R^2	0.0054
Cragg–Uhler (Nagelkerke) R^2	0.0195

3.3 Hosmer–Lemeshow Goodness-of-Fit Test

Table S4. Hosmer–Lemeshow test using $g=10$ risk deciles. Because of the very large sample size, the test inevitably rejects the null of perfect fit. Calibration plots (not shown) confirm that mis-calibration is practically negligible for most deciles.

Statistic	Value
χ^2 (df = 8)	1 855.5
p-value	$< 2.2 \times 10^{-16}$
